# Supplementary material for: The crucial role of adhesion in the transmigration of active droplets through interstitial orifices
Source: Nat Commun. 2023 Feb 25;14:1096. doi: 10.1038/s41467-023-36656-0 (PMC9968312; doi:10.1038/s41467-023-36656-0)
Supplement: Supplementary file 1 — Supplementary Information [file 41467_2023_36656_MOESM1_ESM.pdf]

# The crucial role of adhesion in the transmigration of active droplets through interstitial orifices
# Supplementary information

A. Tiribocchi,[1] M. Durve,[2] M. Lauricella,[1] A. Montessori,[3] D. Marenduzzo,[4] and S. Succi[1,2,5]

[1] *Istituto per le Applicazioni del Calcolo CNR, via dei Taurini 19, 00185 Rome, Italy*
[2] *Center for Life Nano Science@La Sapienza, Istituto Italiano di Tecnologia, 00161 Roma, Italy*
[3] *Department of Engineering, Roma Tre University, Via Vito Volterra 62, 00146 Rome, Italy*
[4] *Scottish Universities Physics Alliance, School of Physics and Astronomy, University of Edinburgh, Edinburgh EH9 3JZ, United Kingdom*
[5] *Department of Physics, Harvard University, Cambridge, MA, 02138, USA*

## SUPPLEMENTARY NOTE 1: NUMERICAL ASPECTS

In this section we provide further details about the computational model and the simulation parameters.

The equation of the order parameter $\phi_1$ (Equation 2 of the main text), the one of the polarization $\mathbf{P}$ (Equation 3 of the main text) and the Navier-Stokes equations (Equations 4 and 5 of the main text) are integrated by using a hybrid lattice Boltzmann (LB) method [1], where the first two are solved via a finite-difference predictor-corrector scheme and the latter through a standard LB approach.

Simulations are run on rectangular boxes, where the vertical direction is kept constant at $L_{\mathrm{z}} = 170$ while the horizontal one $L_{\mathrm{y}}$ ranges between 500 and 700 lattice sites. We set periodic boundary conditions along the $y$-axis and two flat walls along the $z$-axis, placed at $z = 0$ and $z = L_{\mathrm{z}}$. Within the microchannel we place a droplet containing an active gel, whose concentration is described by the scalar field $\phi_1(\mathbf{r}, t)$, kept approximately equal to 2 inside and 0 everywhere else, and whose orientation is captured by the polar field $\mathbf{P}(\mathbf{r}, t)$, initially uniform and parallel to the $y$ direction within the drop ($\mathbf{P}(\mathbf{r}, 0) = \mathbf{P}_{\mathrm{y}}(\mathbf{r}, 0)$, with $|\mathbf{P}_{\mathrm{y}}| = 1$) and zero outside. At the walls of the channel we impose no-slip conditions for the velocity field, i.e. $v|_{z=0,L_{\mathrm{z}}} = 0$, and no wetting for $\phi_1$, i.e. $\phi_1|_{z=0,L_{\mathrm{z}}} = 0$ throughout the simulation. No specific anchoring conditions (such as perpendicular or parallel) are imposed at the walls for the polar field $\mathbf{P}$.

Following the design proposed in Ref.[2], the constriction is represented by two semi-circular pillars placed symmetrically at distance $h$ and attached to opposite walls (see Fig.2 of the main text). Their solid structure is modeled through two auxiliary *static* phase fields $\phi_2(\mathbf{r})$ and $\phi_3(\mathbf{r})$, positive within each pillar and zero outside. In addition, the velocity field is set to zero inside throughout the simulation. Finally, to ensure that their interface remains perpendicular to the flat walls, we impose neutral wetting condition for both phase fields. Although a more accurate modeling of the constriction is possible (using, for example, a full lattice Boltzmann approach [1, 3]), this one combines a good numerical stability with a relatively easy implementation of mesoscale interactions (such as repulsion and adhesion controlled by proper free energy terms, see Eq.1 of the main text) between the active droplet and the pore.

Thermodynamic parameters have been chosen as follows: $a = 0.04$, $k = 0.06$, $M = 0.1$, $\alpha = 0.1$, $\kappa = 0.04$, $\xi = 1.1$, $\Gamma = 1$, $\eta \simeq 1.67$, $\epsilon_{ij,i<j} = \epsilon = 0.1$. Their choice generally ensures a high numerical stability and a reasonable mapping to real physical units (see next section). In particular, the first two values, $a$ and $k$, control the surface tension $\sigma$ of the active droplet, given by $\sigma = \sqrt{8ak/9} \simeq 0.045$, while $\alpha$ fixes the values of $\phi_{\mathrm{eq}}$ and $\mathbf{P}_{\mathrm{eq}}$ (see section Methods of the main text) and controls the isotropic-to-polar transition. Also, $\kappa$ and $\xi$ describe the elasticity of the polar liquid crystal, with the latter larger than 1 to account for flow aligning particles. The rotational viscosity $\Gamma$ sets the time scale of the relaxation of the polarization and describes the viscous torque associated with its rotation, while $\eta$ represents the viscosity of fluid, both within and outside the droplet. Finally, an equal repulsion, whose strength is controlled by $\epsilon$, is set between droplet and pillars. Its value is high enough to prevent merging between different phase fields.

Lattice spacing and integration timestep are kept fixed to $\Delta x = 1$ and $\Delta t = 1$. The contractile activity $\zeta$ has been varied between $-10^{-4}$ and $-10^{-3}$. Keeping fixed the thermodynamic parameters defined above, the optimal values of $\zeta$ ensuring a balance between interfacial deformations and splay distortions (guaranteeing a rectilinear motion) range roughly between $-5 \times 10^{-4}$ and $-10^{-3}$. Finally, the constant $\gamma_{ij}$ in Eq.1 of the main text gauges the strength of the adhesion forces. We impose an equal value of adhesion coefficient between drop and each pillars, i.e. $\gamma_{12} = \gamma_{13}$, while other entries are set to zero. Since a constant value of $\gamma$ throughout the pore generally does not yield a complete crossing, we set two different values $\gamma_{\mathrm{L}}$ and $\gamma_{\mathrm{R}}$ of adhesiveness on the half left of the pore (entry) and on the half right (exit) respectively, with $\gamma_{\mathrm{L}} > \gamma_{\mathrm{R}}$, to ensure a transmigration along the positive direction of the $y$-axis. In our simulations they approximately vary between $10^{-3}$ and $5 \times 10^{-2}$, while optimal values generally depend on the previous thermodynamic parameters as well as on the ratio $\lambda = h/D$, where $D$

is the diameter of the droplet. As discussed in the main text, $\lambda$ ranges from 0.2 to 0.8.

## SUPPLEMENTARY NOTE 2: MAPPING TO PHYSICAL UNITS

Here we provide an approximate mapping between simulation parameters and real physical units. Our runs are performed on a rectangular mesh of size varying from $L_y = 500 \div 700$ (length of the channel) to $L_z = 170$ (height of the channel), with lattice spacing $\Delta x = 1$ and integration time step $\Delta t = 1$. A droplet of radius $R = 45$ lattice sites is placed within the microchannel, sufficiently far away from the constriction. Following previous works on self-propelled active fluid droplets [4, 5], we fix the length, time and force scales to the following values: $L = 1\mu$m, $T = 10$ms and $F = 100$nN (in simulation units these scales are all equal to one). Hence, our simulation would approximately correspond to a microfluidic channel of length ranging from 0.5 to 1mm in which an active droplet of diameter $D \simeq 90\mu$m has an effective shear viscosity $\eta_{eff} \simeq 1.5$kPa·s, an effective elastic constant $\kappa \simeq 4$nN, a surface tension $\sigma \simeq 4$mN/m, moving with speed $1 \div 10$ $\mu m/s$ in a Newtonian fluid, such as water. Also, one would have a rotational viscosity $\Gamma \simeq 1$kPa·s, a diffusion coefficient $D_\phi = Ma \simeq 0.4$ $\mu$m$^2$/s and an activity $\xi \simeq 100$Pa. Finally, the drop speed in simulation units varies between $v \simeq 10^{-4}$ and $v \simeq 10^{-3}$, values that keep the Reynolds number $\mathrm{Re} = v\rho D/\eta$ lower than 0.1 and ensure that inertial effects can be neglected. In addition, the capillary number $\mathrm{Ca} = v\eta/\sigma$ ranges approximately between 0.1 and 0.001, thus droplet rupture remains an unlikely event.

## SUPPLEMENTARY NOTE 3: SUPPRESSION OF TRANSMIGRATION IN THE PRESENCE OF ADHESION

As discussed in the main text, the transmigration through sufficiently narrow constrictions is generally inhibited if a constant and uniform value of $\gamma$ is set between droplet and pillars. In Fig.2 of the main text we have shown that the process can actually occur if the adhesion coefficient at the entry of the pore, $\gamma_{\mathrm{L}}$, is larger than the one the exit, $\gamma_{\mathrm{R}}$, provided that $\gamma_{\mathrm{min,L}} \leq \gamma_{\mathrm{L}} \leq \gamma_{\mathrm{max,L}}$ and $\gamma_{\mathrm{min,R}} \leq \gamma_{\mathrm{R}} \leq \gamma_{\mathrm{max,R}}$.

In Supplementary Movie 7 we show an active droplet moving within a microchannel where $\lambda \simeq 0.5$, $\gamma_L = 5 \times 10^{-2}$ and $\gamma_R = 3 \times 10^{-2}$. Note that, with this value of $\lambda$, one has $\gamma_{\mathrm{min,L}} \simeq 2 \times 10^{-2}$, $\gamma_{\mathrm{max,L}} \simeq 5 \times 10^{-2}$, $\gamma_{\mathrm{min,R}} \simeq 5 \times 10^{-3}$ and $\gamma_{\mathrm{max,R}} \simeq 2 \times 10^{-2}$, thus the transmigration is expected to be suppressed, since $\gamma_{\mathrm{R}} > \gamma_{\mathrm{max,R}}$. At the entry of the pore the dynamics is overall akin to that discussed in Fig.2 of the main text for $\lambda \simeq 0.5$. Indeed, portions of the interface, approximately located on opposite sides with respect to the longitudinal midline of the microchannel, adhere to the pillars, enabling the droplet to squeeze in. However, once a large part (more than half) of the droplet has passed the center of the pore, the motion stops and the droplet gets stuck on a peanulike configuration. This occurs because the droplet propulsion is not sufficient to overcome the excess of adhesion (controlled by $\gamma_{\mathrm{R}}$) which, increasing the connectivity between interface and pillars, ultimately prevents the transmigration.

In Supplementary Movie 8 we show the case in which $\lambda \simeq 0.2$, $\gamma_{\mathrm{L}} = 1.5 \times 10^{-2}$ and $\gamma_{\mathrm{R}} = 10^{-2}$. Here $\gamma_{\mathrm{min,L}} \simeq 2 \times 10^{-2}$, $\gamma_{\mathrm{max,L}} \simeq 4 \times 10^{-2}$, $\gamma_{\mathrm{min,R}} \simeq 5 \times 10^{-3}$, $\gamma_{\mathrm{max,R}} \simeq 1.5 \times 10^{-2}$, hence once again the crossing should be inhibited since $\gamma_{\mathrm{L}} < \gamma_{\mathrm{min,L}}$. Indeed, once the droplet hits the pore, the low adhesion forces at the entry (controlled by $\gamma_{\mathrm{L}}$) do not guarantee a prolonged contact between interface and pillars, thus basically impeding the droplet to snake in. The rearrangement of the liquid crystal orientation due to the internal fluid flow drives the droplet downwards and then backwards, causing its detachment from the pore.

As mentioned in the main text we note that the values of the adhesion coefficients depend upon other thermodynamic parameters, such as elasticity of the liquid crystal and interfacial tension, speed of the droplet, viscosity, diffusion constant, repulsion as well as initial position, whose modification would require an adjustment of $\gamma_{\mathrm{L}}$ and $\gamma_{\mathrm{R}}$.

## SUPPLEMENTARY NOTE 4: SMOOTHER ADHESION DESIGN

In the main text we have shown that the transmigration occurs if two different adhesion coefficients, $\gamma_{\mathrm{L}}$ and $\gamma_{\mathrm{R}}$, are set at the entry and exit of the gap, with $\gamma_{\mathrm{L}} > \gamma_{\mathrm{R}}$. More specifically, if the diameter of the pillar is $l$, one has $\gamma = \gamma_{\mathrm{L}}$ for $0 \leq y \leq l/2$ and $\gamma = \gamma_{\mathrm{R}}$ for $l/2 \leq y \leq l$, i.e. the adhesion strength changes sharply following a step function. A smoother variation can be obtained by assuming that $\gamma(y) = 0.5[(\gamma_{\mathrm{L}} + \gamma_{\mathrm{R}}) + (\gamma_{\mathrm{L}} - \gamma_{\mathrm{R}}) \tanh((-y + y_0)/a)]$, where $y_0$ and $a$ are two constants controlling position and width of the slope around the midline of the constriction. Increasing $a$ progressively augments the width of $\gamma(y)$, thus ultimately diminishing the value of $\gamma$ nearby the midline of the pillars (see Fig.S1). In Supplementary Movie 9 we show, for example, three simulations for $\lambda \simeq 0.5$ and $a = 1$ (left), $a = 15$ (middle), $a = 20$ (right), while other parameters are kept equal to those of Fig.2g-n of the main text. Our results show that the typical dynamic features of a droplet crossing a medium-size constriction are preserved albeit, for increasing values of $a$, the transmigration time gradually augments (see Fig.S2) until the process is completely arrested and the

drop gets trapped within the pore.

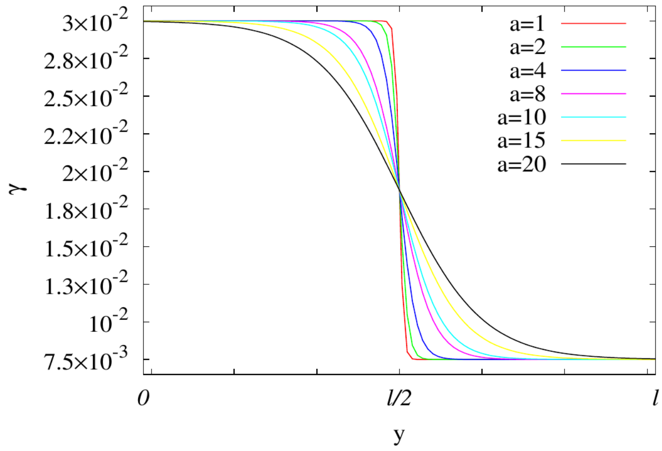


Figure S1. Functional forms of the adhesion strength within the constriction for $\lambda \simeq 0.5$. Here $\gamma(y) = 0.5[(\gamma_L + \gamma_R) + (\gamma_L - \gamma_R)\tanh((-y + y_0)/a)]$, where $\gamma_L = 3 \times 10^{-2}$ and $\gamma_R = 7.5 \times 10^{-3}$, while $y_0$ and $a$ control position and width of the slope. Also, $l$ is the diameter of the circular pillar. Increasing the parameter $a$ augments the width of the curve around the midline of the pillars.

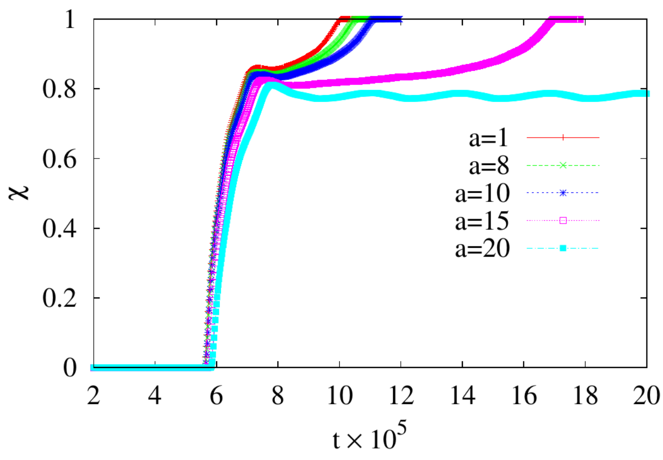


Figure S2. Transmigration order parameter $\chi(t) = A_C(t)/A_T(t)$ for $\lambda \simeq 0.5$ and $a$ ranging from 1 to 20. Other parameters are the same as the ones of Fig.2g-n of the main text. Here $A_C$ is the area fraction of the droplet between the midline (located at $l/2$) and the exit of the constriction (located at $l$), while $A_T$ is the total area of the droplet within the pore.

## SUPPLEMENTARY NOTE 5: THIN FILM-LIKE DYNAMICS

When moving through highly constrained micro-environments, droplets (or cells) can often experience a momentum sink due to the presence of walls placed at very close distance. To account, in a phenomenological way, for this thin film-like dynamics, one can add, to the momentum balance equation, a frictional force of the form $\mathbf{f} = -b\mathbf{v}$, where $b$ is a frictional coefficient and $\mathbf{v}$ is the fluid velocity. This approach is often followed in active gel theory to study systems where the dry limit is of experimental relevance, such as in cells crawling on solid substrates [4]. The effect of this extra term leads to results in qualitative agreements with the ones described in the main text. Indeed, although the frictional force reduces the droplet speed, the crossing is generally guaranteed unless $b$ overcomes a critical drag $b_c$ (whose value depends on the other model parameters) beyond which the transmigration is hindered. In Supplementary Movie 10 we show, for example, two simulations for $\lambda \simeq 0.5$ with $b = 5 \times 10^{-4}$ (left) and $b = 10^{-3}$ (right). Other parameters are the same as those in Fig.2g-n of the main text. While for $b = 5 \times 10^{-4}$ the transmigration shares the same features as those observed in medium size pores, for $b > b_c \simeq 10^{-3}$ the drop gets stuck within the pore, since the speed decrease reduces the propulsion necessary to complete the crossing.

## SUPPLEMENTARY REFERENCES


[1] L. N. Carenza, G. Gonnella, A. Lamura, G. Negro, A. Tiribocchi, Lattice Boltzmann Methods and Active Fluids, Eur. Phys. Journ. E **42**, 81 (2019).

[2] P. M. Davidson, J. Sliz, P. Isermann, C. Denais, J. Lammerding, Design of a microfluidic device to quantify dynamic intra-nuclear deformation during cell migration through confining environments, Integrative Biology **7**, 1534-1546 (2015).

[3] T. Krüger, H. Kusumaatmaja, A. Kuzmin, O. Shardt, G. Silva, E.M. Viggen, *The Lattice Boltzmann Method: Principles and Practice*, Springer International Publishing, (2016).

[4] E. Tjhung, D. Marenduzzo, M. E. Cates, Spontaneous symmetry breaking in active droplets provides a generic route to motility, Proc. Nat. Acad. Sci. USA **109**, 12381 (2012).

[5] E. Tjhung, A. Tiribocchi, D. Marenduzzo, M. E. Cates, A minimal physical model captures the shapes of crawling cells, Nature. Commun **6**, 5420 (2015).
