# Supplementary material for: The crucial role of adhesion in the transmigration of active droplets through interstitial orifices
Source: Nat Commun. 2023 Feb 25;14:1096. doi: 10.1038/s41467-023-36656-0 (PMC9968312; doi:10.1038/s41467-023-36656-0)
Supplement: Supplementary file 3 — Description of Additional Supplementary Files [file 41467_2023_36656_MOESM3_ESM.pdf]

Description of Additional Supplementary Files

File name: Supplementary Movie 1.
Description: This movie shows the transmigration of an active dropet across a constriction where A 0.5 and $= -8 \times 10^{-4}$.

File name: Supplementary Movie 2.
Description: This movie shows the dynamics of an active dropet within a microchannel where A 0.5, $= -8 \times 10^{-4}$ and adhesion is absent. No crossing occurs.

File name: Supplementary Movie 3.
Description: This movie shows the dynamics of an active dropet within a microchannel where A 0.5, $= -10^{-3}$ and adhesion is absent. No crossing occurs.

File name: Supplementary Movie 4.
Description: This movie shows the transmigration of an active dropet with A 0.5, $= -7 \times 10^{-4}$, $\gamma L = 3 \times 10^{-2}$ and $\gamma R = 7.5 \times 10^{-3}$.

File name: Supplementary Movie 5.
Description: This movie shows the dynamics of an active dropet within a microchannel where A 0.5, $= -8 \times 10^{-4}$, $\gamma L = 3 \times 10^{-2}$ and $\gamma R = 3 \times 10^{-2}$. No crossing occurs.

File name: Supplementary Movie 6.
Description: This movie shows the transmigration of an active dropet where A 0.2, $= -7 \times 10^{-4}$, $\gamma L = 2.5 \times 10^{-2}$ and $\gamma R = 10^{-2}$.

File name: Supplementary Movie 7.
Description: This movie shows the dynamics of an active dropet where A 0.5, $= -7 \text{ X } 10^{-4}$, $\gamma L = 5 \times 10^{-2}$ and $\gamma R = 3 \times 10^{-2}$. Here $\gamma R > \gamma max,R$ 2×10^{-2}, thus the transmigration is prevented.

File name: Supplementary Movie 8.
Description: This movie shows the dynamics of an active dropet where A 0.5, $= -7 \times 10^{-4}$, $\gamma L = 1.5 \times 10^{-2}$ and $\gamma R = 10^{-2}$. Here $\gamma L < \gamma min,L$ 2×10^{-2}, thus the transmigration is prevented.

File name: Supplementary Movie 9.
Description: This file contains three movies of an active droplet migrating within a microchannel with A 0.5 and $= -7 \times 10^{-4}$. Here the adhesion strength γ varies as $\gamma(y) = 0.5[(\gamma L + \gamma R) + (\gamma L - \gamma R) \tanh((-y + y0)/a)]$, with a = 1 (top movie), a = 15 (middle movie), a = 20 (bottom movie)

File name: Supplementary Movie 10.
Description: This file contains two movies of an active droplet migrating within a microchannel with A 0.5, = -7×10^{-4}, $\gamma L = 3 \times 10^{-2}$, $\gamma R = 7.5 \times 10^{-3}$. Here the frictional parameter b is introduced, with $b = 5 \times 10^{-4}$ (left movie) and $b = 10^{-3}$ (right movie).
